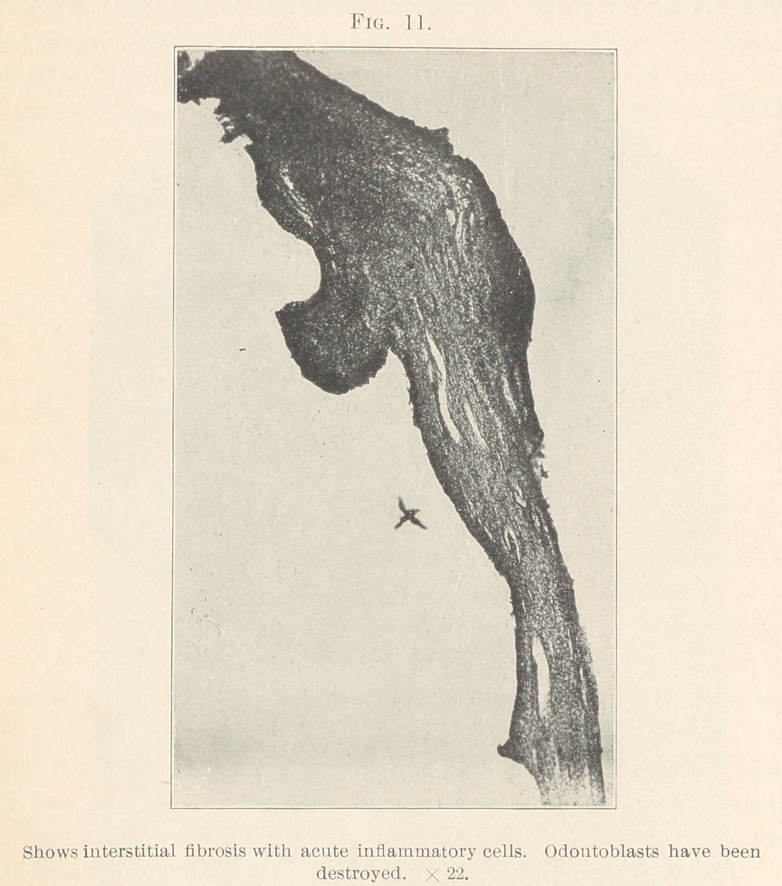# Pulp Degeneration

**Published:** 1904-10

**Authors:** Eugene S. Talbot

**Affiliations:** Chicago


### International Dental Journal.

Vol. XXV. October, 1904. No. 10.

### Original Communications.[1]

### PULP DEGENERATION.[2]

BY EUGENE S. TALBOT, M.S., D.D.S., M.D., LL.D., CHICAGO.

There are two forms of pulp degeneration,—physiologic and pathologic. The physiologic is along the line of evolution and under the general law of economy of growth or use and disuse of structures. Physiologic degeneration was discussed in a paper, "The Evolution of Pulp."[3] It was shown that structures nourishing the placoid scales were larger than the scales themselves. Later, in some sharks, toothed birds, elephants, etc., the circumscribed pulp is as large as the tooth; in the horse and cow it is smaller, while in the anthropoid apes and man the pulp grows smaller and smaller until, in adult life, the apical end is so small that only one or two small arteries and nerves enter the root of the tooth. I demon-

---

[1] The editor and publishers are not responsible for the views of authors of papers published in this department, nor for any claim to novelty, or otherwise, that may be made by them. No papers will be received for this department that have appeared in any other journal published in the country.

[2] Read at the annual session of the American Medical Association, Section on Stomatology, Atlantic City, June 7 to 10, 1904.

This paper is one of a series read before this Section for a number of years, and was referred to in my paper on "The Constitutional Causes of Tooth-Decay."

[3] Journal American Medical Association, August 2, 1902.

strated the vasomotor system of the pulp with nerve-endings in a paper on the "Vasomotor System of the Pulp,"[1] still later in "Constitutional Causes of Tooth Decay."[2] I also demonstrated nerve degeneration and inflammation resulting in abscess of the pulp by disease of the body in connection with the vasomotor system and nerve degeneracy.

A pulp with such a record as I have demonstrated could hardly avoid pathogenic degeneration. Scarcely a pulp is exempt from influences of this, due to diseases of the body, external violence, or pathologic changes. In the very nature of events, physiologic degeneration must necessarily result in pathogenic degeneration under the law of economy of growth and the struggle for existence between organs, influence by bodily defects. Before taking up the different degeneracies, the nature of the pulp must be briefly considered.

The number of nerves, arteries, and veins entering the apical foramina depends on the age of the individual and the tooth itself. A larger number enters early in tooth development than later in life, when the foramina is exceedingly small. Age and exostosis naturally reduce the size of the opening. Only one or two arteries enter the pulp-chamber from the main trunk. These divide and subdivide, forming many branches and loops.

Because of the small opening at the apical end of the root, collateral circulation is impossible; hence, with end nerves and arteries, the pulp is an excellent illustration of an end organ. This constitutes its susceptibility to disease. The pulp enclosed within bony walls is without an opportunity for expansion in arterial dilation and sclerosis; it has only one or two small trunk arteries and veins for supply and waste. The blood likewise increases disease susceptibility. The vasomotor system makes the pulp to respond to any disease to which the general system may be subjected. Diapedesis follows. Thermal changes from without also modify the circulation of the pulp. Sudduth, and later Miller, are of opinion that there are no lymphatics in the pulp. If they be not present, still the pulp has great predisposition to degeneration, since Wedl, Tomes, Smale and Colyer, and many others, as well as myself, have found large spaces, without walls, whose lymphatic nature has not been

---

[1] Journal American Medical Association, December 19, 1903.

[2] Dental Digest, December, 1903.

determined. That débris and waste products may be carried from the pulp through the veins seems probable.

One influence but little considered in relation to pulp degeneration or tooth-structure in general, and one that exerts a marked consequence on tooth-decay, is the factor of interstitial gingivitis, abrasion, and erosion, which are degenerative conditions that take place at the fourth period of stress, at the senile stage or period of evolution at from forty to forty-five years of age. Not infrequently the senile stage occurs prematurely in neurotics and degenerates. At this period all excretory organs are weakening, faulty metabolism results, and the vasomotor system does not respond quickly. Marked disturbances take place in all the structures of the body, including the alveolar process as well as the pulp. Wedl in 1872 first called attention to the senile condition of tooth-structures shown by their discoloration.

Morbid change in the pulp other than nerve-end degeneration, inflammation resulting in abscess, as already discussed, may be summed up as arteriosclerosis, endarteritis obliterans, thrombosis, and embolism, cloudy swelling, fatty degeneration, mucoid, colloid, hyaline, and amyloid degeneration, pulp-stones, neoplasm, and fibroma. Some of these have been discussed by Wedl, Tomes, Smale and Colyer, Hopewell-Smith, Black, Bödecker, Arkövy, Andrews, Römer, Morgenstern, Caush, Latham, and many others, and can be studied more at length in the original monograph.

Here it is not my intention to study each morbid condition, but to show that the pulp is susceptible to them (individually and collectively), resulting in tooth degeneration.

Among vascular changes and circulatory disturbances, thrombosis in the blood-vessels of the pulp is not uncommon. From the present knowledge of pathology and the pathogenic condition of the pulp, it is evident how thrombosis must occasionally result. The pulp, an end organ without anastomosis and collateral circulation, the blood returning through a single vein, creates an anatomic predisposition for formation of a thrombus. The many degenerations and retrogressive changes which take place in the pulp make it susceptible to this morbid state. The spontaneous death of the pulp which sometimes follows disease can be thus accounted for. Formation of different calcic deposits causes the current to become slower and the leucocytes to be retarded in their progress from and to the apical end of the root-canal. In time the blood-plates separate from the blood-current and are caught at the apical end of the pulp-canal. Sudden blindness occurs under similar conditions. The vessels become injured or abnormal, due to calcic deposits, and other retrogressive changes and stasis take place, eventually furnishing a basis for future thrombosis and inflammation (Fig. 1).

Fig. 1.

Thrombosis of capillaries of pulp and inflammation. × 137. Arteries and capillaries closed. Thrombus. Acute inflammation, showing there has been a hyperæmic condition.

A thrombus may be located in any part of the arterial system, but more especially the heart. Simple or septic fragments may become dislodged and carried through the blood-streams to or into the pulp of the tooth. Having entered this cavity, its return is almost impossible.

Embolism consists of various structures, such as fat drops, tissue fragments, tumor-cells, air, etc. These follow the blood-current. The size of the body regulates the distance to which an embolus may travel. It stops in vessels whose lumen prevents its passage. More frequently it is arrested at the bifurcation of the artery. The pulp is especially adapted for this purpose, since it is an end organ, with numerous loops terminating in one or more veins for exit.

Emboli, according to Hektoen, act in two ways, mechanically, clogging the circulation, and specifically, depending on the nature of the embolus, whether infected or sterile, whether composed of dead or living cells, capable of further proliferation. The circulation may be mechanically obstructed. If septic material has lodged in a

FIG. 2.

Dilated vessel. Diapedesis and embolus. × 280.

blood-vessel, inflammation may extend to the surrounding tissues (Fig. 2).

*Endarteritis Obliterans and Arteriosclerosis.*—Inflammation of the arterial coats in the pulp is very common. This is due, in a degree, to pulp embryogeny, anatomy, environment, and to its end-organ nature, as already stated. The diseases most commonly observed are endarteritis obliterans and arteriosclerosis. While it is not uncommon for each coat of the artery to take on a special type of inflammation, yet all frequently become involved.

Endarteritis obliterans is an inflammation of the inner coat of the artery, usually of a chronic type. The inflammation may arise from an irritant in the blood-current from the main current, through the vaso vasorum, or through the lymphatics. The first is the most usual; in the alveolar process all three may occur. In the pulp, irritation in the blood-stream is the most common method. Proliferation of the endothelium results. Bands of fibrous tissue develop. The blood-vessels become obstructed and finally obliterated, impeding the circulation (Fig. 3).

Fig. 3.

The wall in one artery is thickened (endarteritis) and almost occluded by inflammatory products. In the smaller artery the intima contains round-celled infiltration almost occluding it. The pulp-tissues show the myxomatous character very well, branched spindle and round nucleated cells in many places. × 225.

The structure pulp, made up of loops of blood-vessels and situated within bony walls, with only one or two arteries and veins for the passage of blood, renders it a unique end organ, and its arteries susceptible to arteriosclerosis. This, together with endarteritis obliterans, predispose the arteries to degeneration and necrosis. This is a thickening of the arterial walls, especially of the intima. It is secondary, according to Hektoen, to certain inflammatory or degenerative changes in the media. This is seldom observed early in life. It is commonly found after puberty, but more frequently at the senile stage, from forty years on. The causes producing arteriosclerosis in other parts of the body produce it in the pulp arteries.

The causes are usually autointoxication and drugs taken into the system, which likewise become irritants. Besides the distensive force and change in composition of the blood, local irritation on the

FIG. 4.

An enlarged artery in an early stage of thickening, the small vessels plugged up, well-marked myxomatous pulp-tissue. × 225.

arterial wall is an active cause. In diseases such as syphilis, gout, rheumatism, Bright's disease, alcoholism, and chronic mercurial, lead, brass, arsenic, and bromide poisoning, the walls become irritated, resulting in thickening of the arterial coats.

"The inebriate, whose brain and body after death exhibit a confused mass of wreckage, which the pathologist is often unable to trace back to the exact causes and conditions, has, according to Crothers, always sclerotic conditions of the large and small arteries, together with atrophic and hypertrophic states of the heart, kidneys, and liver, with fatty degeneration and calcification of the coats of the arteries. These organic changes are so frequently present in inebriates that they constitute a marked pathology which is traceable to the use of alcohol."

These irritants, acting through the vasomotor system and increasing the arterial pressure, finally cause paralysis and diminution of the caliber of the arteries and capillaries, producing stasis of blood (Fig. 4). This morbid state of the arteries tends to produce any or all of the other degenerations previously referred to.

FIG. 5.

Pulp-stones scattered throughout, here and there a form of round-celled infiltration, longitudinal nerve-trunks, few degenerated vessels surrounded by hyaline degeneration in the middle of nerve-trunk. Early sclerosis and cloudy swelling or granular degeneration. Odontoblasts *in situ*. × 21.

The inflammatory process of the intima was first charged to direct irritation of material floating in the blood. Rokitansky and Thoma are of opinion that it is secondary and dependent on the degenerative changes of the middle coat. This view I cannot accept, since autointoxic states produce irritation in the blood-streams.

Many degenerations of the pulp are the result of arteriosclerosis, endarteritis obliterans, and nerve degeneration. These degenerations occur in connection with each other; in other words, sometimes two, three, and even more are to be found in the same pulp. The causes producing these degenerations are not understood.

*Retrogressive Changes.*—One direct result of arteriosclerosis and endarteritis obliterans is cloudy swelling and fatty degeneration. These conditions are observed in connection with such diseases as typhoid fever, septicæmia, and other acute infections and toxic diseases. The tissues present a whitish or shiny appearance, without fibrous structures. Under the microscope the tissues present an opaque mass and do not take stain. The cells are quite large and swollen (Fig. 5).

"When a tissue, as for instance the heart-muscle, receives a diminished quantity of blood on account of the narrowing of the

Fig. 6.

Fatty degeneration, acute pulpitis, sclerosis of nerves. Nerve degeneration, dilatation of vessels, faint outline of degenerated odontoblasts. × 137.

lumen of the arteries due to thrombosis, embolism, or disease accompanied by thickening of the intima, albuminous and fatty changing, remarks Hektoen, usually result. In the case of the different forms of anæmias, degenerations with fat production are found in the liver, heart, kidneys, and muscles. In such conditions there is not enough oxygen and other nutritive material to maintain the function of the cells. In actual starvation there is first absorption of all the fat in the body, accompanied by a marked diminution of the structure. In the later stages, albumin and fatty degeneration take place. Albuminal and fatty changes are very common in febrile diseases. They occur in practically infectious diseases and in a large number of the intoxications, such as the drug poisons. They are also found in abnormal metabolism, due to direct action of poisons and the abnormal process of oxidation." Owing to the pulp's peculiar structure and environment, fatty degeneration is commonly found in its tissue (Fig. 6).

Amyloid degeneration is a peculiar degeneration of the connective tissue, causing an albuminous substance to be deposited in

Fig. 7.

Shows pulp-stones and their close relation to the vascular channels. Dilated vessels with amyloid deposit. × 62.

the surrounding tissue. The walls of the blood-vessels also become involved. It presents a shiny appearance and differs from other tissues in that it turns a dark red color with iodine. The morbid state is found in syphilis, tuberculosis, chronic dysentery, etc. (Fig. 7). Almost every structure in the body may be involved.

Hyaline degeneration (Fig. 8) is, according to Stengle, closely allied with amyloid, mucoid, and colloid degeneration, and all can

Fig. 8.

Calcareous deposit, medullary nerve. Early connective cell formation. × 225.

pass into each other. It can occur in tissues during infectious and septic processes, following traumatism, in autointoxications such as drug poison, hemorrhages, in cicatrices, in senile blood-vessels, arteriosclerosis, endarteritis obliterans, and in the nervous system. It can also occur in connective tissue which has undergone a change by inflammation. This morbid state depends for its action on local or general nutritive disturbances. The pulp, therefore, is susceptible to it. The intima, as well as the entire walls of the small blood-vessels in the pulp, easily becomes involved. Some investigators believe that fat connective tissue cells so arrange themselves as to undergo a change into myelin substances (Fig. 9). These ultimately lead to calcification. This raises the question of calcic deposits or so-called pulp-stones. Pathologists know that tissues elsewhere in the body (which have necrosed or degenerated) are the localities where lime salts are deposited. Dying tissue which has undergone more or less change possesses, according to Ziegler, a kind of attraction for the lime salts in solution in the body. The tissues, to which attention has been called, are especially susceptible

Fig. 9.

This shows medullary nerve-fibres and internodes, axis-cylinders, myelin degeneration. × 280.

to calcic changes; hyaline and fatty degeneration, tissues involved in disease or drug poisoning, already mentioned here and elsewhere. Regions affected by slight degeneration and in structures like the pulp, a constricted end organ, are predisposed to deposits of lime salts. Calcic deposits have different shapes and location in the pulp-tissue. Circumscribed structures which appear solid under the microscope, to the naked eye, or to the touch, are not pulp-stones or calcic deposits, but in a large percentage of cases belong to other retrogressive changes. These deposits (Fig 10) are, no doubt due to degeneration of pulp-tissue, especially in structures undergoing hyaline or fatty degeneration. Large masses of deposits in the form of spherules often occur. Bone formations are sometimes observed.

Fig. 10.

Shows medullary nerve-fibres slightly thickened. The connective tissue is degenerating, and hyaline odontoblasts show well on both surfaces. × 156.

These deposits, both in pulp-stones and spherules, take on a dirty, bluish-violet color, with hæmatoxylin. These Dr. Latham and I have observed many times. Crystals may sometimes occur.

"This applies, however, as Ziegler remarks, only to deposits of lime carbonates and phosphates, and not to those of lime oxalate." These deposits may take place at any time, but are most likely at the senile or fourth period of stress.

I shall not consider neoplasm at length in this paper, since Dr. Latham has this subject under discussion, but will now refer to fibroid degeneration in closing. Fibroid growth of the pulp may be both rapid or slow. Inflammatory reaction in fibrous pulps is rare, although when followed by infection or exposure, it may take place. Various degeneracies like those already mentioned are liable to occur, especially those in which connective tissue in general is predisposed. The fibres are observed in bundles, closely packed together, with many connective tissue corpuscles shown at intervals. Fibroid degeneration is easily distinguished from the other degeneracies of the pulp (Fig. 11).

Fig. 11.

Shows interstitial fibrosis with acute inflammatory cells. Odontoblasts have been destroyed. × 22.

In these cases, the blood-vessels and nerve-tissue are relatively few. The blood-vessels remaining usually have thickened walls, especially in the external and middle coats. This, of course, narrows the lumen. Not infrequently the blood-vessels are entirely obliterated. These fibromas, very common in exposed pulps, are not now under consideration. In nearly if not all of these degenerations the blood-vessels are first involved, later nerve-tissue.

All these degenerations, including the pathologic processes of evolution, are the direct constitutional causes of tooth-decay, erosion and abrasion brought about by diminution of tooth vitality.

## Figures and Tables

**Fig. 1. f1:**
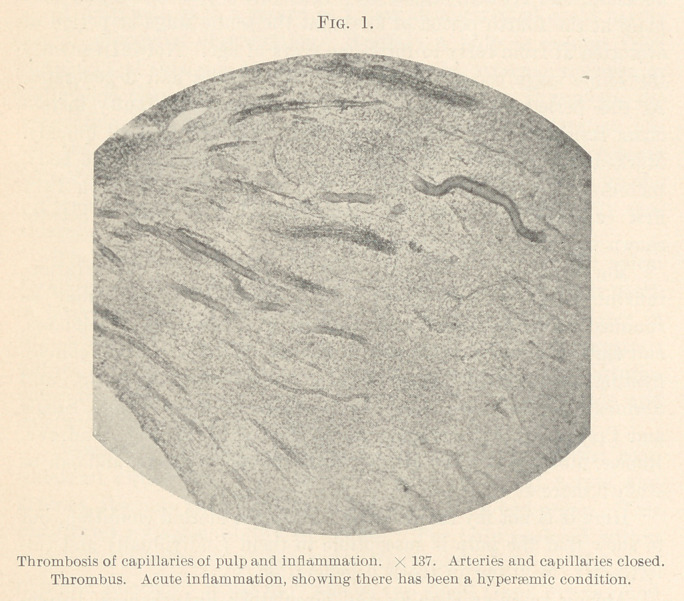


**Fig. 2. f2:**
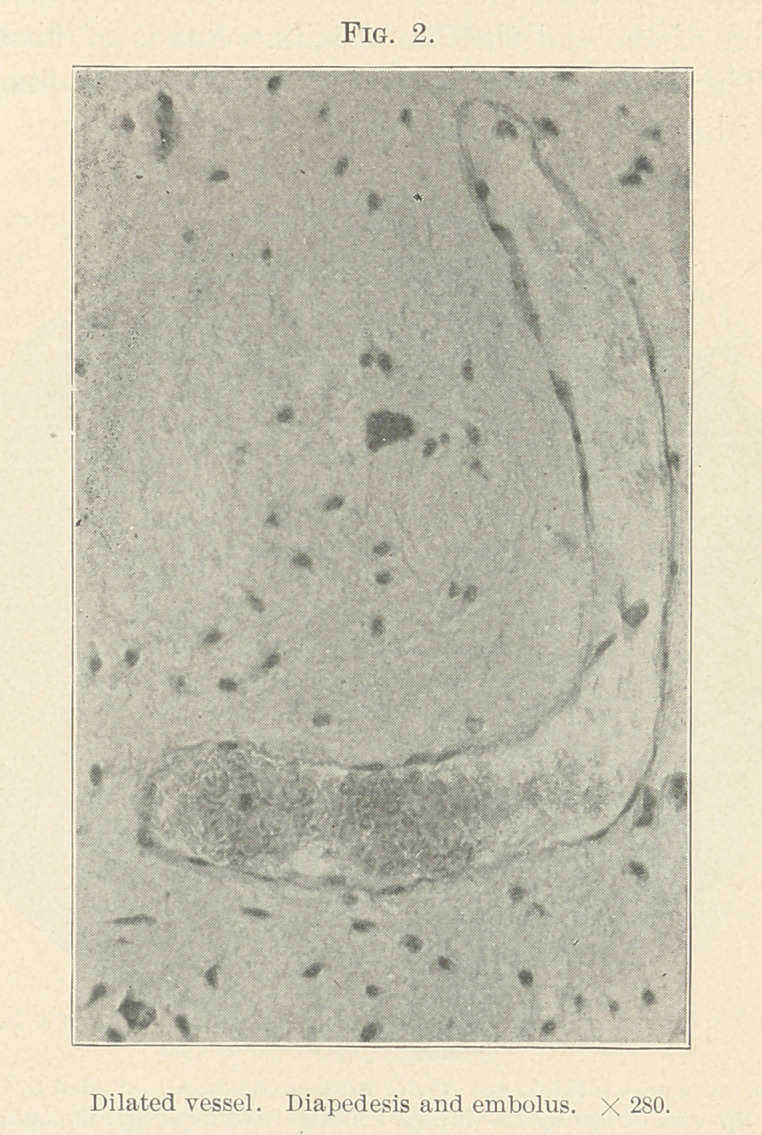


**Fig. 3. f3:**
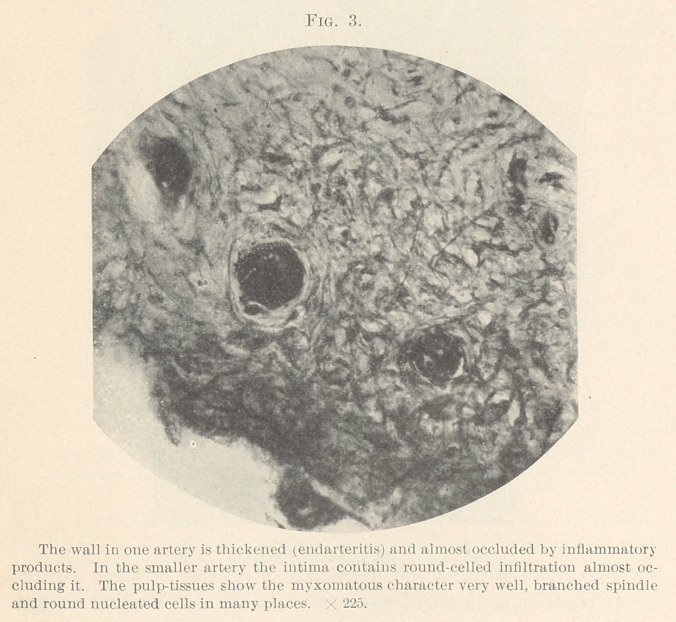


**Fig. 4. f4:**
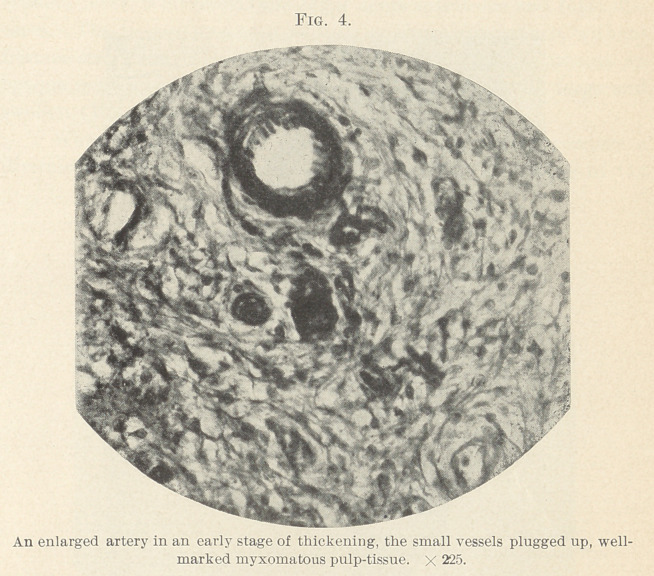


**Fig. 5. f5:**
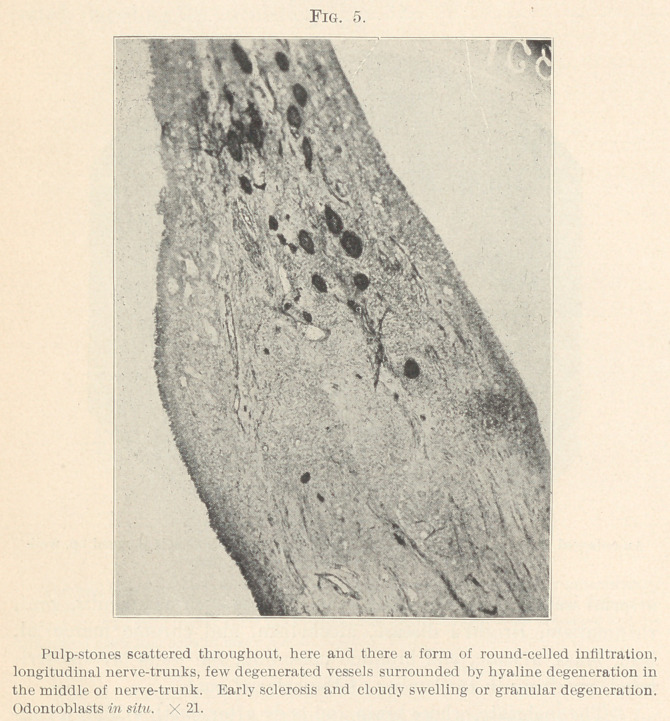


**Fig. 6. f6:**
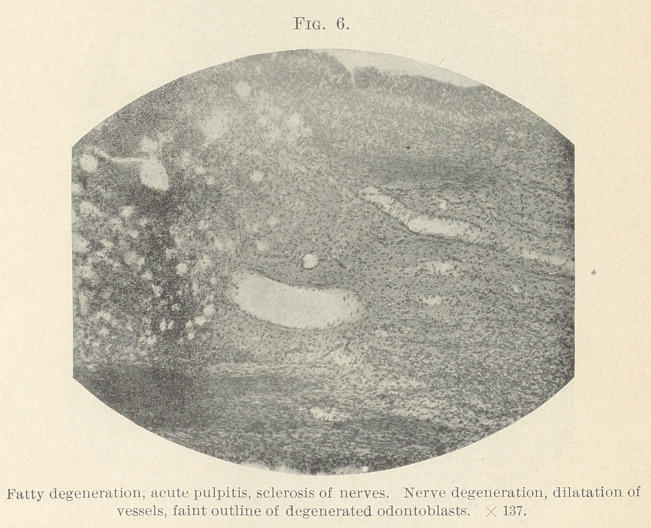


**Fig. 7. f7:**
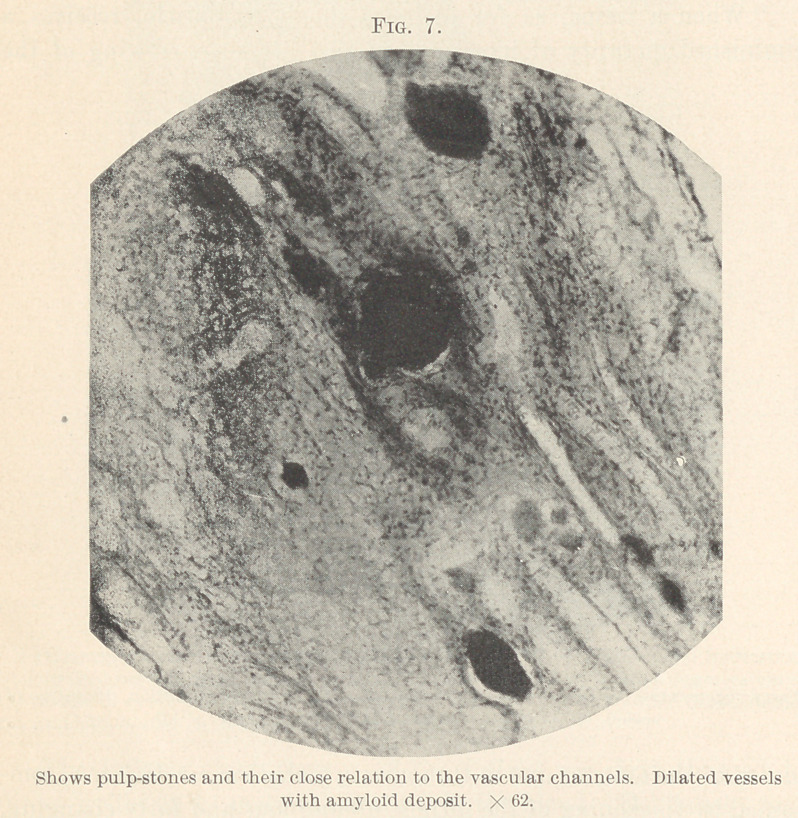


**Fig. 8. f8:**
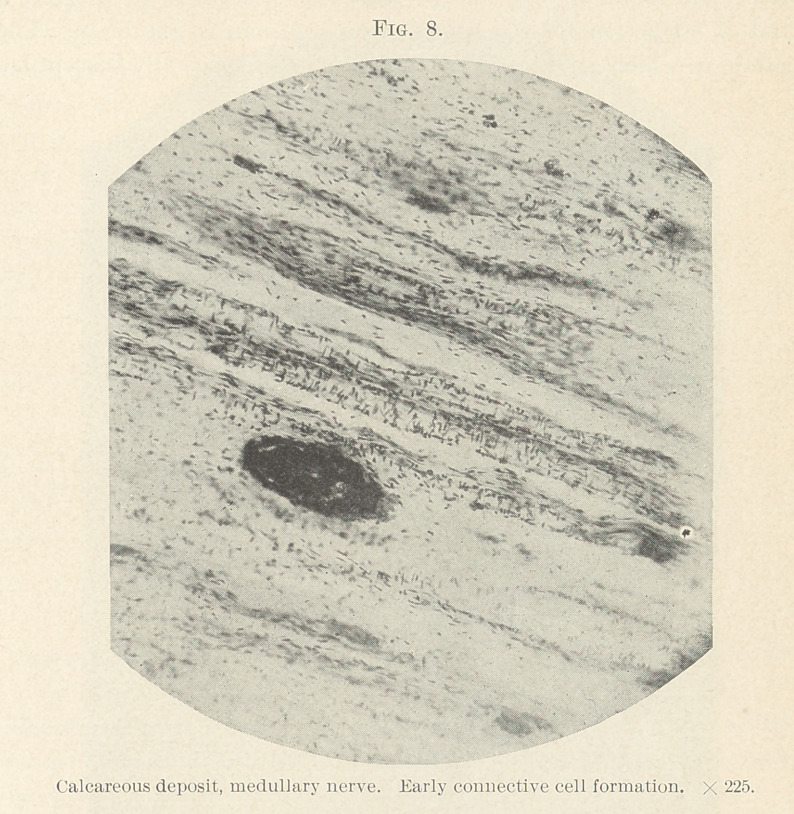


**Fig. 9. f9:**
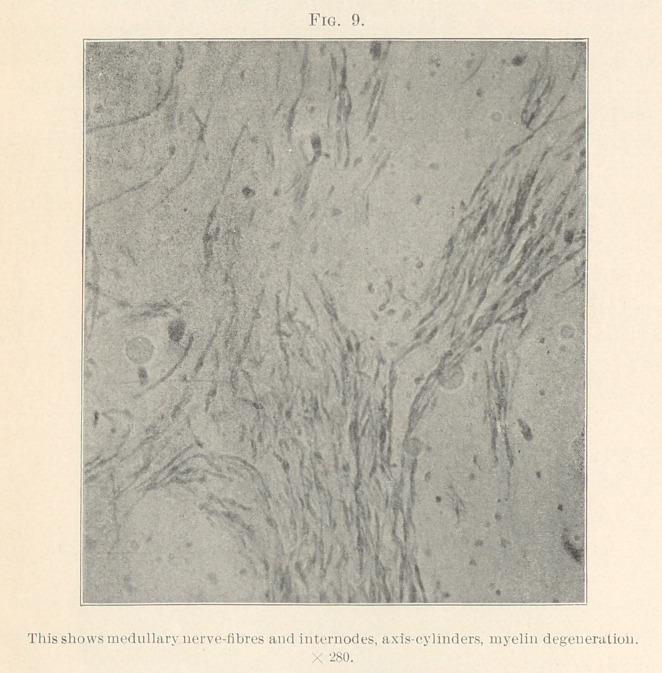


**Fig. 10. f10:**
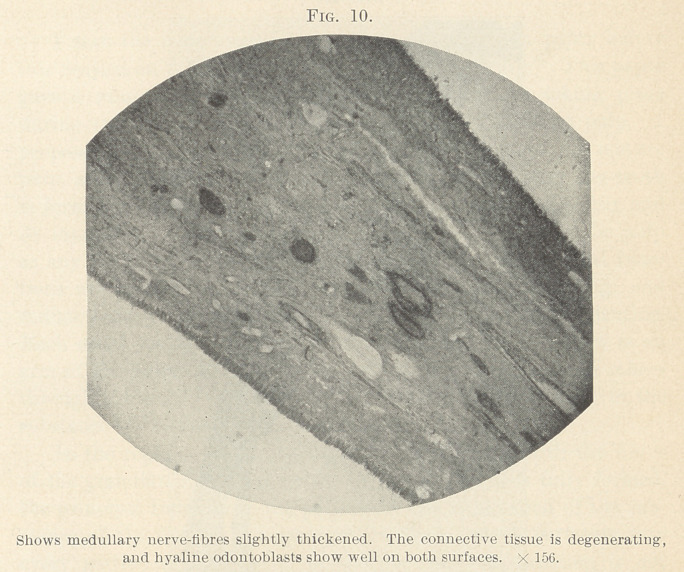


**Fig. 11. f11:**